# Supplementary material for: Metamodelling of a two-population spiking neural network
Source: PLoS Comput Biol. 2023 Nov 30;19(11):e1011625. doi: 10.1371/journal.pcbi.1011625 (PMC10688753; doi:10.1371/journal.pcbi.1011625)
Supplement: S1 Supplementary Section — (PDF) [file pcbi.1011625.s001.pdf]

S3 Hierarchical Partial Least Squares Regression

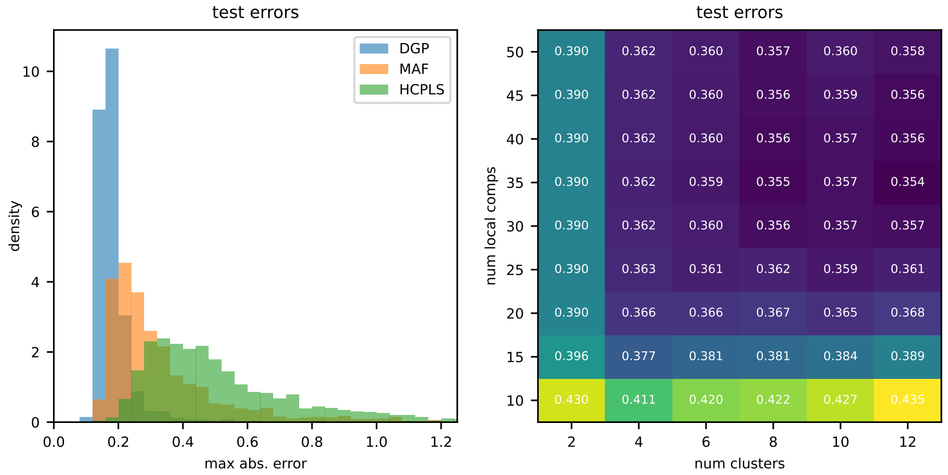

The left-hand side of the figure shows the distribution over maximum (over frequencies) errors on the test data set for all three metamodels. The right-hand side shows the results from the hyperparameter search for the HCPLSR metamodel, for two global components.

In addition to the DGPR and MAF metamodels, we also trained a Hierarchical Partial Least Squares Regression (HCPLSR) model [1]. HCPLSR is an extension of Partial Least Squares Regression (PLSR) [2], which has gained popularity in chemometrics [3]. HCPLSR has also previously been used for metamodelling of different biological dynamical systems [4, 5].

In HCPLSR, multiple PLSR models are deployed in different regions of the parameter space, increasing the flexibility of the model. A global PLSR model is first applied, on which fuzzy C-means clustering (FCM) [6, 7] is performed on either the x-scores or y-scores. A separate local PLSR-model is subsequently trained on each cluster, and the final prediction is made by a weighted sum of the local model predictions, with the weights given by the probability of belonging to the separate clusters as determined by the fuzzy clustering.

We trained a HCPLSR metamodel on the same data set as the DGPR and MAF metamodels. A basis expansion of the metamodel inputs (i.e. parameters of the network model) were done to include second order terms. A hyperparameter search was done over the number of global components, number of local components and number of clusters. The left-hand side of the figure above shows the distribution over test errors of

all three metamodels. The HCPLSR model is less accurate than both the DGPR and MAF metamodels, indicating that it is not sufficiently flexible compared to the other two metamodels. The right-hand side of the figure shows the test errors achieved with different numbers of local components and the number of clusters. The best results were achieved with two global components. In terms of computational cost, the HCPLSR is significantly faster than both other metamodels, and evaluating the model at 50 locations takes only a few milliseconds.

References

1. Tøndel K, Indahl UG, Gjuvsland AB et al. Hierarchical Cluster-based Partial Least Squares Regression (HC-PLSR) is an efficient tool for metamodelling of nonlinear dynamic models. BMC Syst Biol 5, 90 (2011). doi: https://doi.org/10.1186/1752-0509-5-90

2. Wold S, Martens H, Wold H. The multivariate calibration problem in chemistry solved by the PLS method. Matrix Pencils. Lecture Notes in Mathematics, vol 973. Springer. 1983. doi: 10.1007/BFb0062108

3. Wold S, Sjöström M, Eriksson L. PLS-regression: a basic tool of chemometrics. Chemometrics and Intelligent Laboratory Systems. 2001;58:109–130

4. Tøndel K, Martens H. Analyzing complex mathematical model behavior by partial least squares regression-based multivariate metamodeling. WIREs Computational Statistics. 2014;6:440–475

5. Tøndel K, Vik JO, Martens H, Indahl UG, Smith N, Omholt SW. Hierarchical multivariate regression-based sensitivity analysis reveals complex parameter interaction patterns in dynamic models, Chemometrics and Intelligent Laboratory Systems. 2013;120:25–41

6. Bezdek J. Pattern Recognition With Fuzzy Objective Function Algorithms. Kluwer Academic Publishers. 1981. doi: 10.1007/978-1-4757-0450-1.

7. Berget I, Mevik BH, Næs T. New modifications and applications of fuzzy C-means methodology. Computational Statistics & Data Analysis. 52;2403–2418. 2008. doi: 10.1016/j.csda.2007.10.020
