# Supplementary material for: Metamodelling of a two-population spiking neural network
Source: PLoS Comput Biol. 2023 Nov 30;19(11):e1011625. doi: 10.1371/journal.pcbi.1011625 (PMC10688753; doi:10.1371/journal.pcbi.1011625)
Supplement: S2 Supplementary Section — (PDF) [file pcbi.1011625.s002.pdf]

S1 hyperparameter scans

For both the DGPR and MAF metamodels, a hyperparameter grid search was run in order to find suitable hyperparameters. The training procedure was the same for both models. Optimization was done using the Adam optimizer, and and the loss function was computed for the full training and test data sets every other epoch. If the loss function on the test data set had not improved by a value more than 1 in 20 epochs, training was terminated. The model was saved every time the loss function on the test data set improved.

DGPR

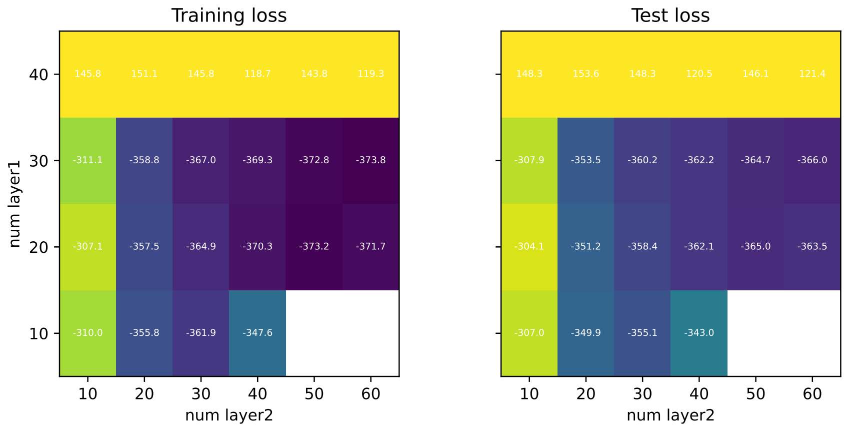

Fig A. Loss values for different hyperparameter values of the DGPR metamodel. White squares are NaNs.

For the DGPR metamodel, the number of GPs in the first layer and the number of latent GPs in the second layer were varied. The number of layers were found based on trial and error, where we found that 1 layer performed worse, likely due to lack of expressiveness, and 3 layers or more also performed worse, likely due to increased optimization difficulties. The number of inducing points were kept at 128 for each layer, it was found that increasing it did not improve the model significantly. Each model was trained with learning rates of 0.01 and 0.001. Figure A shows the results for the DGPR metamodel. The best model from either of the learning rates are shown. Based on this, the model with 20 GPs in the first layer and 50 GPs in the second layer was chosen.

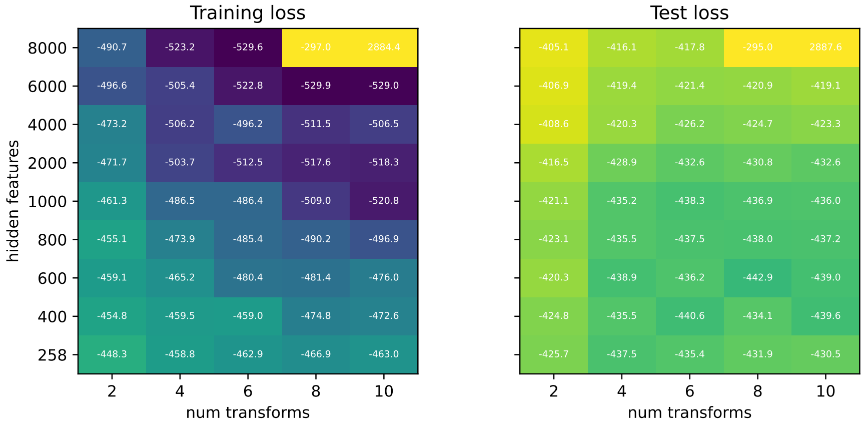

Fig B. Loss values for different values of hyperparameters of the MAF.

MAF

For the MAF, number of transforms, the number of hidden features in the MADE block, and whether a dropoutrate of 0.5 was applied or not, were varied. Each model was trained with learning rates of 0.001 and 0.0001. Figure B shows the best outcome for each combination of hidden features and number of transforms.
