# Supplementary material for: Metamodelling of a two-population spiking neural network
Source: PLoS Comput Biol. 2023 Nov 30;19(11):e1011625. doi: 10.1371/journal.pcbi.1011625 (PMC10688753; doi:10.1371/journal.pcbi.1011625)
Supplement: S3 Supplementary Section — Description and results from HCPLSR metamodel. (PDF) [file pcbi.1011625.s003.pdf]

S2 full posterior plots

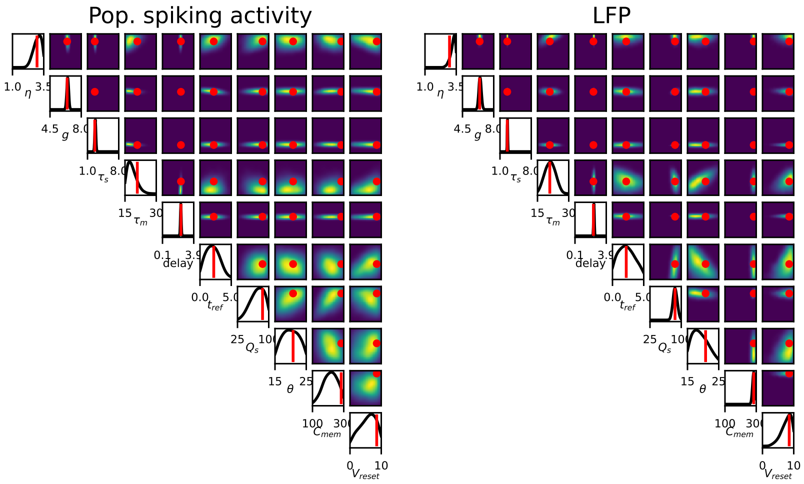

Full posterior distributions from which the subset in Fig 5 in the main text is taken.
