# Supplementary material for: Metamodelling of a two-population spiking neural network
Source: PLoS Comput Biol. 2023 Nov 30;19(11):e1011625. doi: 10.1371/journal.pcbi.1011625 (PMC10688753; doi:10.1371/journal.pcbi.1011625)
Supplement: S4 Supplementary Section — (PDF) [file pcbi.1011625.s004.pdf]

S4 Validation of LFP computation scheme

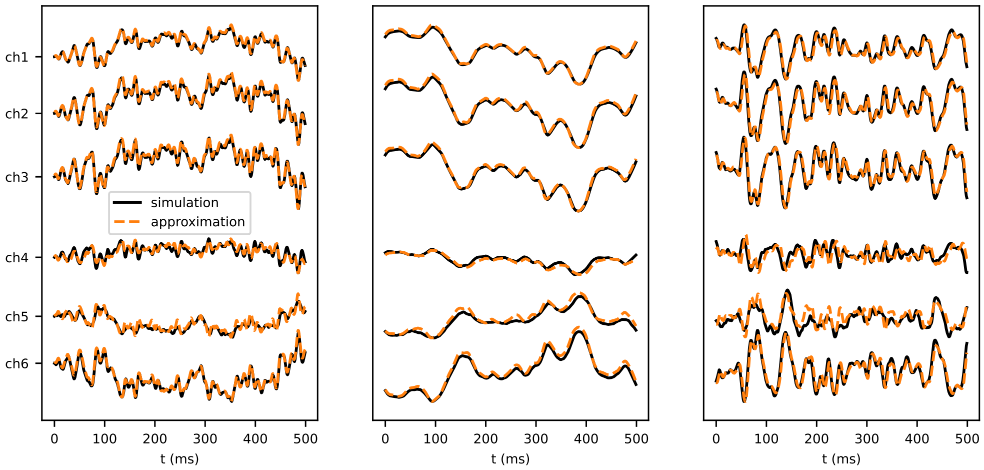

Three example simulations of the LFP using the full hybridLFPy scheme (black lines) and the approximation (orange dashed lines).

In order to validate the LFP computation scheme, we ran a full simulation using hybridLFPy for 3 different simulations, and compared the results. The figure above shows three examples in which the full hybridLFPy scheme was run and compared with our approximation scheme. The parameters were drawn from a uniform prior on the same domain as the main data set used. There are some deviations, particularly in channel 5, but the approximation is generally very good.
