# Supplementary material for: Metamodelling of a two-population spiking neural network
Source: PLoS Comput Biol. 2023 Nov 30;19(11):e1011625. doi: 10.1371/journal.pcbi.1011625 (PMC10688753; doi:10.1371/journal.pcbi.1011625)
Supplement: Attachment — Submitted filename: metamodel_response2.pdf [file pcbi.1011625.s006.pdf]

Dear Editor,

We hereby resubmit our manuscript PCOMPBIOL-D-22-01359 “Metamodelling of a two-population spiking neural network” by Jan-Eirik W. Skaar, Nicolai Haug, Alexander J. Stasik, Hans Ekkehard Plesser, Gaute T. Einevoll and Kristin Tøndel for publication as a research article in PLOS Computational Biology.

We thank the editor and the reviewers for their time and efforts, as well as new suggestions for improvements to the manuscript.

In particular, we have addressed your request to “state clearly what advances your work provides and what is the significance of your study for the computational neuroscience field” in several ways. In our view the main advances of our work to the computational neuroscience field are:

- Metamodelling, i.e. approximating the outputs of an expensive simulator and using the approximation for scientific inquiry, has not seen much use in computational neuroscience. We utilize metamodeling techniques on the much studied Brunel-network, comprising one inhibitory and one excitatory population of leaky integrate-and–fire neurons. We apply metamodeling techniques, and show that the probability distribution over the power spectral density of the local field potential (LFP) and population spiking activities from the Brunel network in the asynchronous irregular firing regime can be accurately approximated based on roughly 1000 simulations. Depending on the metamodel, generating new approximations to the simulations is on the order of a thousand times faster than running the simulator itself. We compare a deep Gaussian process regression (DGPR) model, a multi-layered model based on Gaussian processes, to a Masked Autoregressive Flow (MAF) model, which is commonly used in density estimation tasks. We find that for this network the DGPR metamodel generates predictions of the population spiking activities that are more accurate than the commonly used MAF model.

- Trained DGPR models can be used in combination with experimental data, that is, spikes or LFP, to predict model parameters of the underlying neural network models generating these signals. This approach is illustrated in the present paper for the Brunel network where the analysis revealed which model parameters are most decisive (“sensitive”) for determining the output spike rate and LFP for the network. The same analysis also reveals how accurately the underlying model parameters in principle can be determined from measurements of spikes or LFPs. A concrete observation resulting from the present analysis is that observing the LFP signal more accurately can constrain some of the parameters of the underlying Brunel model compared to the population spiking activities. In particular the membrane capacitance and the scale of the synaptic strength are significantly more constrained by the LFP compared to the population

spiking activities, while for the remaining 8 parameters investigated, both signals are roughly equally informative.

- We find that for the task of inverse modeling, i.e. determining the probability distribution over simulator parameters given an observed simulator output, the more accurate DGPR metamodel also gives more accurate posterior distributions, i.e. the probability distribution over parameters given an observed simulation output, compared to the commonly used MAF metamodel.

- Metamodels based on Gaussian processes are highly flexible and offer a framework that has been underutilized, and can be useful for many other computational biologists, both in neuroscience and other subfields. We thus think the demonstrated success of DGPR when applied to the Brunel network should be of interest for many readers of the journal. We think the successful use of a metamodel offering a large computational speedup with little loss of accuracy for a network model is of particular relevance since the computational requirements when simulating large-scale networks will remain a bottleneck for the foreseeable future.

To better highlight these advances provided by our paper we have now changed that last paragraph in the abstract to the following

Specifically, we apply and compare two different metamodelling techniques, masked autoregressive flows (MAF) and deep Gaussian process regression (DGPR), to estimate the power spectra of two different signals; the population spiking activities and the local field potential. We find that the metamodels are able to accurately model the power spectra in the asynchronous irregular regime, and that the DGPR metamodel provides a more accurate representation of the simulator compared to the MAF metamodel. Using the metamodels, we estimate the posterior probability distributions over parameters given observed simulator outputs separately for both LFP and population spiking activities. We find that these distributions correctly identify parameter combinations that give similar model outputs, and that some parameters are significantly more constrained by observing the LFP compared to observing the population spiking activities.

We change the last two sentences in the author summary to the following

In this study, we train and compare different metamodelling techniques, data-driven approximations that are much faster to run, to two different signals generated by a two-population recurrent network model, the population spiking activities and the local field potential (LFP). Further, we invert the metamodels, and demonstrate that it can reliably find the different combinations of parameters that can give rise to an observed simulation output. We compare the accuracy of the metamodels on both the forward and inverse problem, and

investigate to what degree the parameters are constrained by observing the two different signals.

We change the final paragraph in the introduction to the following

We show that the simulator can be accurately captured by metamodels trained on around 1000 example simulations, and that for the present situation, the DGPR metamodel is able to more accurately estimate the complex function described by the simulator, compared to the MAF metamodel. We find that posterior distributions estimated from the DGPR metamodel is more accurate compared to the ones estimated from the MAF metamodel, but takes longer to compute. We estimate the posterior distributions separately for DGPR metamodels trained on the population spiking activities and the LFP, for a variety of different simulation outputs, and find that some parameters are significantly more constrained by the LFP compared to the population spiking activities, while for others both signals are roughly equally informative. We also investigate the pairwise correlations of the posterior distributions over the domain on which the metamodels were trained.

We change the final two paragraphs in the conclusion to the following

The DGPR metamodel performed better than the MAF metamodel, but at the cost of higher computational demands for training and evaluation of log probabilities. The DGPR metamodel can be evaluated on the order of 1000 times faster than the simulator and the MAF metamodel on the order of 100 times faster than the simulator. The trained metamodels can be used to estimate posterior distributions over simulation parameters given observed simulation outputs. The posterior distributions estimated from the DGPR metamodel had higher marginal variances and lower posterior predictive variances compared to the posterior distributions estimated from the MAF metamodelthat the DGPR metamodel produces. This indicates that the DGPR metamodel produces more accurate posterior distributions compared to the MAF metamodel. When dealing with complex models, utilizing DGPR for forward modelling may therefore be a good alternative, particularly when the amount of data is a limiting factor, and the increased training time is not a problem.

Based on the estimated posterior distributions, the LFP was found to contain significantly more information about the synaptic strength and membrane capacitance, while for the remaining eight parameters, both signals were equally informative.

We address the points raised by the reviewer in detail below.

Reviewer #1: Recommendation
The authors have made diligent efforts to account for feedback in the previous round, which they should be commended for. This manuscript now very clearly lays out the role of

metamodeling with respect to state-of-the-art techniques used in computational neuroscience for SBI - namely the normalizing flows (MAFs) used to estimate likelihoods (forward model) for inference as in SNLE. SNPE is primarily used in favor of SNLE, so I find this paper's focus on the metamodel (estimation of the likelihood/forward model) to be a refreshing re-examination of the utility of such approaches for models with expensive simulations with intricate emergent phenomena like the Brunel model. However, I struggle to identify an aspect of the advancements/insights laid out in this paper that meet the publication criteria of PLoS Computational Biology. I recommend rejecting this paper for the reasons elaborated below.

We thank the reviewer for the positive comments regarding the improvements in the manuscript, and for the thorough review.

Novelty/innovation/insight
In the first review, I stated my opinion that this manuscript could meet the publication criteria for this journal along the lines of innovation/insight if they did either one of two things: a.) demonstrate benefits of metamodeling with respect to SoTA approaches from of SBI or b.) generate foundational insights about the Brunel model using these techniques. Showing that a metamodel can be used to approximate the Brunel model to a reasonable degree (which they have definitely done) is not enough. While they have made considerable progress towards each of these aims, they don't present enough evidence of a.) methodological advancement or enough analyses to yield b.) foundational insight generated with this methodology.

a.) methodological advancement
Since the original manuscript had a focus on using metamodels towards parameter inference, the authors followed recommendations from Reviewers 1 and 2 to explore the methodological tradeoffs between metamodeling and methods in SBI. Since metamodeling (estimation of the likelihood) is a process within SNLE, the authors accurately point out that the process undertaken in the manuscript is like running SNLE for 1 round. This underscores the fact that metamodeling isn't novel in computational neuroscience, however it is certainly underutilized, and they will be making a case for its use in this paper.

The methodological comparisons in the latest manuscript focus on the approximating model of the Brunel model - the type of metamodel. Indeed they show some evidence that deep gaussian processes are a better approximation than MAFs. for the Brunel model, and also that HCPLSR lacks the expressivity necessary for this problem (I appreciate the inclusion of this in the manuscript knowing how great a focus this was in the original version). Even if this claim that DGPR was a more effective method of metamodeling than MAF for the Brunel model was firmly established (which I'm not entirely sure it is, see section Rigor/completeness), this relative advantage of DGPR would need to be demonstrated for at least two neural circuit models (not just one) to establish that it is in fact a better practice for the field moving forward – thus constituting an advancement worthy of publication.

We would like to point out that we do not claim that DGPR metamodels generally are better than MAF metamodels, but rather that they are an alternative with benefits and drawbacks. We also certainly do not wish to say that DGPR metamodelling is in general a better practice than

other types of models that are in use. Different models will always have a place, and in many cases multiple models can be appropriate, and we would recommend trying different model alternatives.

We discuss the particular trade-offs, strengths and drawbacks of the models we have compared in the final paragraphs of the first section of the Discussion.

After this first round of revision, I would have expected the authors to take a more focused approach on simulation count/computational efficiency – what is the trade-off space (simulation count and/or computational cost vs inference/estimation quality) of HCPLSR or DGPR metamodels vs established methods in SBI.

We have now added more details on the computational cost of simulations/metamodels, see comment below.

b.) foundational insight
The authors certainly made a more concerted effort to expand on the meaning of the posteriors inferred via the metamodel, however I don't believe any concrete insights have been revealed in this manuscript. There are certainly paragraphs in Section 3.4 and Discussion along these lines, but they don't rise beyond descriptions of the visualized/quantified correlations in the posterior, or 1D marginal variances, with some prose about how these parameters relate in an information theoretic context.

To meet publication criteria, we'd want to gain some interpretable insight from inferred posteriors - stipulate a hypothesis(es) from this insight - and then confirm them in simulation. That is not done here.

The fact that conditioning on LFPs vs spiking activity yields less posterior variance is nice to see, but I'm not sure this is surprising or has generated any new understanding of the Brunel model and its function.

In our view, the point of our paper is not to gain a new understanding of the Brunel network, there are already more theoretical frameworks doing just that, but rather to explore empirical methods for metamodeling in computational neuroscience, how it can be used to infer parameter values, and the information carried in LFP versus spikes.

Rigor/completeness
Some of this manuscript is well written and mature, but I found several parts unclear.
- Figure 3 - No mention or explanation is given for why MAF is missing from the right side of the figure.

We agree this can be made clearer.

Currently, the figure caption mentions it as

B: Same as A, but for the LFP. Only the DGPR metamodel was trained on the LFP.

We now additionally write in the first paragraph of the Results section

We compare the accuracies of the forward predictions of all three models on the population spiking activities. The DGPR metamodel is trained on both the LFP and the population spiking activities in order to evaluate the differences in information in the two signals.

- How many parameters are used in ideal DGPR model vs ideal MAF model? Can this explain the overfitting mentioned in the main text?

In the best MAF model, there are roughly 9.5 million parameters, versus roughly 1.3 million for the best DGPR model. This likely also influences the overfitting, but even for much smaller MAF models, there is more overfitting present. We believe it is likely caused by the fact that the optimization target is different between the two models. The nature of gaussian process regression models allows them to be trained by maximizing (an approximation to) the marginal log likelihood instead of the log likelihood.

- Figure 4 - train and test labels are switched (otherwise something is very wrong).

Thanks for catching this, it was indeed a mistaken label in the figure script.

- Are these training errors evaluated on the progressively increasing training set, or the complete dataset irrespective of the amount used in training?

They are evaluated on the increasing training set, not the largest training set we use. The test data set is always the same, regardless of the number of samples we use for training.

- How many simulations were used to build the metamodels used in the following analyses? 1000 because its the elbow you designated?

We used the full 8000 samples for training for all other analyses in the paper.

We add the following to the beginning of section 3.2

For all other experiments, the metamodels used were trained on the full 8000 example simulations.

- What's the relative computational cost (training time per epoch etc.) of fitting these metamodels to simulation cost? I don't have an idea of what the true bottleneck in this parameter inference problem is given the methods you're exploring.

We agree that this is useful information, and now expand on this in Section 3.2. We extend the last paragraphs to the following:

In terms of computational cost, evaluating the log probability of 50 example outputs takes around 4 seconds with the DGPR metamodel, and 0.014 seconds with the MAF metamodel.

Generating 50 samples from the metamodels takes 1.6 seconds for the MAF metamodel, and 0.07 seconds with the DGPR metamodel. By contrast, running a single simulation took on average 4.4 CPU-minutes.

The training time for the metamodels depends on the model parameters and learning rate, as well as the random seed. A full epoch of the 8000 training examples took 4.4 seconds for the DGPR metamodel, and 1.4 seconds for the MAF model. Generally, the training converged after a few hundred epochs for both models, giving a training time on the scale of an hour for the DGPR model and around a third of that for the MAF model.

We also add the following sentences to the discussion

The time it takes both to train and evaluate the metamodels will depend strongly on the available hardware. GPU acceleration makes it significantly faster, and solely using CPUs would significantly increase the time of training/evaluating the metamodels.

- Figure 5 - Which metamodel was used in this figure? I assume DGPR, but it's not explicitly stated.

You are right, this is the DGPR model. We now mention this explicitly in the figure text.

- Figure 5B - no explanation of what x and o are in this figure.

The legend has now been added correctly.

- Figure 5D should have x-axis labels indicating the difference of left vs right.

This is now added.

- Figure 6
- I agree that Figure 6D using the mean (or median) error of the posterior predictive wrt to the conditioning simulation is an appropriate evaluation method for the inference techniques shown here, but I disagree with the interpretation of Figure 6B, and the statement L466-469 about the posterior predictive variance.

- The posterior predictive distribution will reflect the uncertainty of the approximate posterior. A method that underestimates the posterior variance, will have lower posterior predictive variance, and that certainly doesn't make it a better method than one accurately capturing the posterior variance.

We believe you may have possibly misread the sentence. We agree fully that a higher posterior predictive variance does not imply a worse model of the posterior distribution. However, what we state is that the combination of a lower posterior variance (Figure 6A) and a higher predictive posterior distribution (Figure 6B) indicates a worse posterior distribution.

I.e., for the DGPR model, we have higher posterior variance, as well as a lower posterior predictive variance, which should indicate a more accurate posterior distribution.

Reviewer #2: Thanks to the authors for constructively engaging with my comments. The new experiments, especially the comparison with MAF, are quite interesting and now an additional contribution the paper makes. I'm happy with the manuscript as it is and very excited to see it in publication.

Reviewer #3: The authors have satisfactorily addressed all my comments.
