# Supplementary material for: Metamodelling of a two-population spiking neural network
Source: PLoS Comput Biol. 2023 Nov 30;19(11):e1011625. doi: 10.1371/journal.pcbi.1011625 (PMC10688753; doi:10.1371/journal.pcbi.1011625)
Supplement: Attachment — Submitted filename: PLOS_metamodel_paper_response.odt [file pcbi.1011625.s005.odt]

Dear Editor;
We hereby resubmit our manuscript PCOMPBIOL-D-22-01359 “Metamodelling of a two-population spiking neural network” by Jan-Eirik W. Skaar, Nicolai Haug, Alexander J. Stasik, Hans Ekkehard Plesser, Gaute T. Einevoll and Kristin Tøndel for publication as a research article in PLOS Computational Biology.
We thank the editor and reviewers for their time and effort and are grateful for their useful comments and suggestions put forward to improve the manuscript. We have followed most of them, as described below, and we indeed think that the manuscript is substantially improved as a consequence. We also added a new co-author (Nicolai Haug) to help with the new analyses.
The largest changes to the manuscript are the following:
We train a Masked Autoregressive Flow (MAF) on the same task, and compare the Deep Gaussian Process Regression (DGPR) and MAF models on both the forward and inverse modelling tasks. 
In order to find good representations of each model, we do hyperparameter scans for both, which we include in a supplementary section. As both the MAF/DGP perform better than the HCPLSR model, we move the HCPLSR results to a supplementary section. As this more directly relates the DGP metamodel to models commonly used in SBI, we hope this also makes the paper more interesting for those who are using SBI methods. The MAF metamodel generally suffered more from overfitting compared to the DGP metamodel, so we also ran more simulations such that the full data set now consists of 10 000 examples, compared to 4 000 before.
The methods section has been made simpler, in particular the section on Gaussian processes. Additionally, we removed the section on the HCPLSR model from the methods, and moved the results from the HCPLRS to the supplementary section. Figure 1 and 2 are removed, and Figure 3 is moved to the introduction (now Figure 1), in order to give the reader an early overview of the paper. Figures 5, 6, 8 and 9 (now Figures 3, 4, 6, 7), have been updated with the MAF metamodel instead of the HCPLSR. Figure 7 has been integrated into Figure 5 (now Figure 3).  A new figure has been added (Figure 6) that compares the posterior distributions resulting from the DGPR and MAF metamodels.
We have also improved the LFP computation scheme to fully account for all parameters. The largest effect of this is that the membrane capacitance becomes significantly more constrained by observing the LFP. 
A Supplementary section has been added, which includes 4 subsections:
Describing the hyperparameter scans run for the DGPR/MAF metamodels
Plots of the full posterior distributions from which a subset is shown in the Figure 5 (previous Figure 8)
Results from the HCPLSR model
Validation of the LFP computation scheme


We address the points raised by the reviewers in detail below.
Reviewer #1: Skaar et al. present a thorough application of state-of-the-art metamodeling techniques to a highly-relevant and well-studied neural circuit model in theoretical neuroscience: leaky-IF spiking E/I ("Brunel") model. They accurately point out, that metamodeling -- the practice of approximating f(x | theta) or p(x | theta) for expensive simulation-defined models and then leveraging those approximations for scientific/theoretical inquiry -- is underutilized in the field of computational neuroscience. They explain the computational efficiency benefits of Hierarchical Clustering-based Partial Least Squares over alternative metamodeling approximation methods like deep gaussian process regression (GPR) and compare their application to the metamodeling of power spectral density of the Brunel model given 10 parameters (synaptic strengths, voltage, capacitance and time constant parameters of the model). Finally, they show that when executing simulation-based inference with their metamodels (as likelihoods) using MCMC, the spiking activities and LFPs result in different manifolds of degeneracy in the neural circuit model. I commend the authors on the degree of detail and clarity in the manuscript.
We thank the reviewer for this positive assessment.
The work presented is a rigorous application of metamodeling techniques to the Brunel model, and advocates for a well-reasoned position that metamodeling techniques offer a practical, methodologically sound alternative to popular approaches in simulation-based inference. However, the findings presented do not reach the publication standard at PLOS Comp Bio.
- a.) Without showing any methodological comparison to prominent techniques like those referenced in the paper for simulation-based inference (SBI) (ABC sampling, SNPE, SNLE, etc), the work offers no evidence of a computational benefit from using either metamodeling (either GPR or HCPLS). We're also not given a sense of how the method scales with parametric dimensionality (important for neuroscience). After all, the ultimate reason for approximating a high-fidelity simulation-based neural circuit model would be to use that approximation for inversion/parameter inference. Otherwise, the contribution is that simulation cost is reduced by coarsening the fidelity of the neural circuit model simulation, which is not stated as a fundamental aim of this manuscript.
- b.) If this manuscript lacks methodological comparisons to SBI, then one would expect the methodology presented to at least generate some foundational insights about the neural circuit model being analyzed. The insights generated regarding the Brunel model are minimal, and only elaborated upon in descriptive terms. It is certainly interesting that the two types of conditioning yield different manifolds of degeneracy, but what does that mean about the model -- or about the physiological neurons they model. Can you make a novel prediction from this methodologically-generated insight and confirm it with model simulations?
I would be happy to re-read a manuscript, which has been re-written around a firm conclusion regarding lines a or b (or both).

Regarding line of work a.) a good place to start would be to insert the metamodel as an approximation of the simulation process in ABC and MCMC, and demonstrate the speed/accuracy trade-off. In what regimes (e.g. parameter count, simulation senarios (dt, NE, NI) etc.) of SBI does metamodeling offer computationally efficient, high-fidelity model inversion. To push the envelope, you could insert the metamodel into the relevant places of SNLE/SNPE (as you allude to) and do a similar analysis.
Thank you for the detailed response. We are following up on your point a, and have done new analyses, including comparisons to Masked Autoregressive Flows (MAFs), a conditional distribution estimator commonly used in SBI. We compare both forward and inverse models based on the Deep Gaussian Process Regression (DGPRs) model and the new MAF models. We do extensive testing of different hyperparameters for both forward models, the result of which is presented in a new Supplementary section 1. We further compute the posterior distributions resulting from each metamodel on the same 100 examples from the test data set. We show that the posterior resulting from the DGPR metamodel is wider, and that the simulations run with parameters sampled from the DGPR posterior distribution (posterior predictive samples) are closer to the ground-truth, and that the DGPR is a competitive model not only as a forward model, but also in the framework of SBI. We have added a new figure (Figure 6) comparing the posterior distributions obtained from both metamodels. The rest of the figures have been updated with the new results from the MAF metamodel. 
We hope the inclusion of the MAF metamodel will make the paper more interesting also for readers who are using various SBI techniques, as we show that the DGP metamodel can be a good alternative to other likelihood estimators such as MAF in the context of SNLE. We have also changed the wording and made clearer the relationship with SNPE/SNLE and ABC in the Introduction and the Discussion. We now write in the Introduction: 
Estimating this posterior distribution by running simulations, without direct access to the model likelihood has been the focus of work such as Approximate Bayesian computation (ABC) [9, 10], Sequential Neural Posterior Estimation (SNPE) [11, 12] and Sequential Neural Likelihood Estimation (SNLE) [13]. SNPE directly models the posterior, typically as a normalizing flow parameterized by a neural network, while SNLE models the likelihood, and infers the posterior in a separate step. As in SNLE, the present work focuses on creating an approximation to the likelihood, and can thus also be used to infer posterior distributions in the same manner. These methods can also be focused on estimating the posterior probability p(θ|x0 ) for a specific observed output x0 , and generating a local model of the posterior distribution around that point by iteratively running simulations where the posterior density is believed to be high. This approach can be viewed as a metamodel that is trained locally around a specific point of interest in the parameter space. In this case, the metamodel will not have to be as flexible as in the case where one is interested in a much larger volume of the parameter space.
And in the Discussion:
Methods such as Approximate Bayesian Computation (ABC) and Simulation-based Inference (SBI) can also be used to estimate posterior distributions over parameters given specific observed model outputs. Our work is closely related to SBI, particularly SNLE []. The approach in the present work can be considered the same as SNLE run for a single round, where we use the DGP as a likelihood estimator. Thus, in cases where one is interested in estimating posterior distributions, particularly in cases where one has limited data and model flexibility is a concern, DGP metamodels may be of use in the SNLE framework. Methods such as Sequential Neural Variational Inference [] for estimating the posterior distribution instead of MCMC sampling can also readily be used. 

We would also like to clarify our motivations a bit: we consider both forward modelling and inverse modelling to be of interest, so our motivation for using metamodels in general is not only in terms of inverting them, but also potentially to provide low-cost alternatives to actual simulations. It’s not particularly important for this network, but for larger networks we think it is something that can be of practical use. We also make this clearer in the discussion.


Reviewer #2: In this work, Skaar and colleagues construct metamodels of the classic Brunel spiking neural network to do fast emulation (i.e., simulate with the metamodel instead of the real simulator) and parameter posterior inference. They perform several thousand simulations of the Brunel network of excitatory and inhibitory populations with 10 free parameters drawn from a Latin hypercube prior, compute the power spectrum of the population rate, as well as PSD of the LFP, which is simulated in a separate hybrid-scheme model using just the spike times. The set of 4000 pairs of {network parameter, PSD} datapoints are used as training data for two types of metamodels, GP regression and hierarchical cluster-based PLS regression, to learn a fast emulator of the forward simulation. They compare the accuracy of the metamodel output against a held-out test set of simulations and find good performance for both, while GPR outperforms HCPLSR. With GPR, the authors then do model inversion to infer network parameters given an observed PSD (again, simulation from a held-out test set). They find reasonable parameter recovery performance and discover parameter correlations in the posterior distribution, while the posterior predictive simulations match that of the original ground-truth simulations. They derive insights about parameters of the Brunel network based on the inference results, such as the scaling between synaptic strength and time constant, which was dependent on which representation of the data (i.e., PSD of the pop rate vs. LFP) one uses as the observation, which in general affected the inferred posterior distribution, e.g., variance of the 1D marginal distribution for some parameters.
We thank the reviewer for the positive remarks.
In general, I personally find this work very interesting and valuable, and is a great addition to the thread of works the group produces towards modeling / inverting network parameters from observed data. As far as I can tell, the work is rigorously done, though some clarifications can be made regarding hyperparam choices, and also the high-level motivation for model choices (i.e., HCPLSR, expanded below). One of the main concern I have is regarding using the hybrid scheme for LFP simulation with fixed parameters. In addition, the manuscript is a bit dense at points, and some visual presentation can be improved. I have a lot of comments below, which are only loosely ranked from high- to low-level comments (my apologies), but they are all meant to improve the clarity of the presentation or strengthen the arguments made in the paper.
- just to clarify, for the LFP simulations, are the hybrid / biophysical network parameters fixed for all simulations, i.e., do all LFP simulations use, for example, a membrane time constant of 20 ms, as implied in Table 4? tIf so, isn’t this problematic that the spiking network simulations are run with variable membrane time constants drawn from the prior, while the LFP is computed with a different and fixed time constant for all simulations? This may also explain why some parameters are more constrained when conditioned on the LFP spectra. Given that the spiking simulations are run anyway, wouldn’t it be straightforward to just compute the LFPs based on another approximation scheme (e.g., Mazzoni et al. 2015), which would presumably be a much better approximation of the LFP for each individual network. I think this is an important point to discuss, and possibly warrants further experiments, at least for a subset of the simulations, to show that the “true” LFP computed from currents in the spiking network (which have variable parameter values) match the LFP from the hybrid scheme (which has fixed parameter values), or that this does not influence posterior inference. Related to this, are PSDs from all 6 LFP channels used for inference? If not, the computational overhead of computing 1 LFP should be minimal?
Thank you for pointing this out, this is indeed a limitation in our methodology. In our initial approach, all parameters apart from the membrane time constant and membrane capacitance were fully accounted for, but you are right that not accounting for the time constant and membrane capacitance could indeed affect the posterior distributions obtained from the LFP.
To improve this, we have modified our LFP modelling scheme such that it correctly accounts for all parameters. We further validate the scheme by running the full scheme for a few different parameter sets and show that our approximation is good, also for the membrane parameters.  In the new Supplementary section 4, this validation is shown. The new posterior distributions from this new scheme turns out to be very similar to the old ones for all parameters apart from the membrane capacitance, which is now significantly narrower for the posteriors conditioned on the LFP.
We have updated Table 4 to include the parameter ranges used, and updated section 2.2 to reflect the changes.
- one thing that was not clear to me at all is the motivation behind using HCPLSR in this work. It is limited in expressivity while introducing computational overhead in the clustering step (as opposed to just doing nonlinear regression), does not allow model inversion, which is a main feature of the GP metamodel, and in general performs worse than the GP (though I suppose the latter point was only discovered by actually running the experiment). Given the group’s previous work (i.e., Skaar, Stasik et al 2020), I would have expected this comparison to be at least against a feedforward CNN model (or some kind of nonlinear regression model in general), which is probably equally fast for emulation time on modern computing hardware, but without loss of expressivity. In general, I think the results regarding HCPLSR is good to know, but does not at all add to the overall story, and can probably be moved to supplemental material given that the methods section is already quite technically challenging (which would also save Fig 2 to highlight a more important result). Alternatively, the authors can provide more motivation for trying HCPLSR (instead of a variety of other equivalent or superior regression techniques) , especially since they do not take advantage of the low dimensional representation found in PLS, as it would be interesting to see if the loadings for different clusters would correspond to qualitatively different regimes of network dynamics, etc.
To clarify our motivations a bit: We consider both forward modelling and inverse modelling to be of interest, so our motivation for using metamodels in general is not only in terms of inverting them, but also potentially to provide low-cost alternatives to actual simulations. It’s not particularly important for this network, but for larger networks we think it is something that can be of practical use. As you mention, PLSR produces low-dimensional representations of the data, which is useful for visualising e.g. differences between different regions of the parameter/output space, and for sensitivity analysis. This becomes increasingly important when the size of the networks increases. Hence, our original motivation for using HCPLSR was that it has been used for metamodelling of other types of biological systems, and utilized for e.g. sensitivity analysis.
We have now trained a Masked Autoregressive Flow (MAF) metamodel in addition to the Deep Gaussian Process Regression (DGPR) metamodel. This should make our work more comparable to the most common approaches taken in typical works utilizing SBI. We do a hyperparameter scan for bothe the DGPR and MAF metamodels. Since both the MAF and DGPR perform significantly better than the HCPLSR model, we agree that using the HCPLSR model is superfluous, and have removed the methods section on HCPLSR, and moved the HCPLSR results to the new Supplementary section 3.
- related to the previous point, it was emphasized in several places that the low dimensional representation in GP is a feature that affords more interpretability, but this was never taken advantage of in downstream analyses. I’m not saying this has to be added to this paper (though it would be very interesting to see!), but without actually looking into the GP latents, it undermines the key motivation for using it, which opens up the question of why not use a more expressive model. Similarly, given the previous results in Skaar, Stasik et al, it would have been natural to use a probabilistic model like mixture density network instead of a CNN to directly learn the posterior over parameters, with the advantage of bypassing the MCMC step as well as eliminating the utility of the GP’s smoothness. This is a rather general comment that can be  addressed in writing of the introduction or discussion, or by performing additional analyses (of GP latents) or testing additional models (a more flexible replacement for the GP, or MDN for direct posterior estimation, etc.), and I leave it to the authors to address it in how they see fit.
For the DGPR metamodel, the main motivation behind using the latent variables was actually not to provide any interpretable factors, but rather to introduce correlations between output channels and to save computation time. One can in principle use independent GPs for each output channel, but that means 1 separate GP per output channel, and a computationally more demanding model. Since GPs are inherently quite costly, keeping the number low is beneficial. 
As also commented above, we are not necessarily only interested in estimating parameters, but also in forward models, which is why we have not included any direct approximations of the posterior distribution. We hope that with the inclusion of the MAF metamodel, in which the setup is very similar to a “standard” SBI setup for posterior estimation, this concern is also addressed.
- L121-131 implies that the prior bounds were restricted to eliminate parameter regions that result in the SR state, but wouldn’t that also exclude parameters that could generate AI dynamics? In the original Brunel phase diagram, this would correspond to the fact that the phase transition between SR and AI state has a slant in the 2D parameter space, while restricting the prior is akin to drawing a proper rectangle around only a part of the AI regime, and I would imagine this effect (i.e., boundaries not parallel to the coordinate axes in the parameter space) would only be exacerbated in the 10-D model here. A discussion of this would be warranted, esp since the authors attempt to capture a wide range of the model’s parameter space with the metamodel. A simple alternative would be to simulate from a broader prior, and post hoc throw out simulations in unwanted regimes (which the authors already do anyway).
We agree, and have added the following text to the discussion of the simulations:
This will also have excluded some AI-like simulations in the vicinity of the boundary between AI and SR, but we consider the difference (starting at $g=4.5$ instead of $g=4.0$) to be sufficiently small as to not significantly diminish the utility of the models.
- regarding the references to SBI methods (in the intro and discussion, L633-640), and in particular, SNPE/SNLE: while it’s true that the sequential version of these algorithms target posteriors for a specific observation, when run just for a single round, they have the exact same properties as the metamodels proposed here in that they are accurate over the full domain of the mechanistic model. In fact, SNLE run for a single round is exactly analogous to the GPR approach here, where the learned emulator/metamodel can be sampled from to produce fast simulations, but just replacing the gaussian process with another conditional density estimator, often of the neural network variety (e.g., normalizing flow). This fact does not diminish the novelty or how interesting the current work is, but is a point that should be clarified (and perhaps practical differences between estimators can then be further discussed, e.g., computational cost of GP vs. flows). Also, on L688: the metamodels plugged into SBI or ABC still produces a posterior that has to be MCMC sampled (as the authors do here), so I’m not sure what this statement is meant to convey?
We agree that this is a point that needs to be clarified. We have changed the paragraph in the introduction to the following: 
Estimating this posterior distribution by running simulations, without direct access to the model likelihood has been the focus of work such as Approximate Bayesian computation (ABC) [9, 10], Sequential Neural Posterior Estimation (SNPE) [11, 12] and Sequential Neural Likelihood Estimation (SNLE) [13]. SNPE directly models the posterior, typically as a normalizing flow parameterized by a neural network, while SNLE models the likelihood, and infers the posterior in a separate step. As in SNLE, the present work focuses on creating an approximation to the likelihood, and can thus also be used to infer posterior distributions in the same manner. These methods can also be focused on estimating the posterior probability p(θ|x0 ) for a specific observed output x0 , and generating a local model of the posterior distribution around that point by iteratively running simulations where the posterior density is believed to be high. This approach can be viewed as a metamodel that is trained locally around a specific point of interest in the parameter space. In this case, the metamodel will not have to be as flexible as in the case where one is interested in a much larger volume of the parameter space.


We have changed the paragraph in the discussion to the following:
Methods such as Approximate Bayesian Computation (ABC) and Simulation-based Inference (SBI) can also be used to estimate posterior distributions over parameters given specific observed model outputs. Our work is closely related to SBI, particularly SNLE []. The approach in the present work can be considered the same as SNLE run for a single round, where we use the DGP as a likelihood estimator. Thus, in cases where one is interested in estimating posterior distributions, particularly in cases where one has limited data and model flexibility is a concern, DGP metamodels may be of use in the SNLE framework. Methods such as Sequential Neural Variational Inference [] for estimating the posterior distribution instead of MCMC sampling can also readily be used. 
- as a reader, the methodological description of GPR was extremely dense to read, while the technical details (and the current Figure 1) don’t contribute to the overall goal of the paper, especially since there’s no comparison of performance as a function of GP hyperparameters. I think most of this can be moved to supplemental materials, to make it easier for a non-technical reader to digest, though this is just my opinion. Similarly, I think the current figure 3 serves as a much better figure 1, as it provides an overview of the study, and should be highlighted as early as possible, instead of technical details about prior samples for the GP or schematic for PLSR. On that note, given the observation that the metamodel output PSD differ in smoothness from the actual simulations, maybe it’s worth exploring different length scales of the GP, as well as discussing the specific choice of hyperparameters (e.g., 10 and 14 GPs in layer 1 and 2, why?)
We have rewritten the section on GPR, and the figure has been removed. Additionally, we  include a hyperparameter scan for both the DGP and MAF, included in supplementary section 1.
Just to clarify, length scales of the GPs are optimized during training.
- some discussions of applying this approach to real neural data (and the limitations) may be of interest to a broader neuroscience audience
We have added the following paragraph to the discussion: 
For experimental data, metamodelling may be of interest in different aspects. In the context of SBI,  metamodels can be used as likelihood estimator in order to find parameters fitting network models to observed experimental data, in a similar way that has been done in this work. This would require larger and more realistic network models than the one used here, but the principle is the same. In the context of forward modelling, metamodels could be applied to experimental data to model effects of experimental parameters and their effects on experimental outcomes. How well this would work in practice, however, is not known. It would require training to specific experimental settings, and could be an interesting direction of future research.


- does the latin hypercube prior outperform a simple uniform prior? Is there a practical or theoretical difference? This is just for my own curiosity.
We suspect that it doesn’t make that much of a difference compared to just regular uniform sampling in our case. We employ it just as an extra caution.
- I think the first paragraph of section 3 (L327-334) is a fantastic summary of the work, and (some version of this) can be restated earlier in the paper as it really helped me as a reader to orient myself in the paper.
We have rewritten the final paragraphs of the introduction, as well as moved Figure 3 to the introduction. This should hopefully make it clearer from the beginning what we are modelling.
- are raw PSDs or log PSDs used as input for all the regression models (and at evaluation)? I’d imagine using the log PSD would help for the regression tasks given the 1/f.	
We use the log. We have added this explicitly to the end of section 2.1, and write
The metamodels are trained on the base 10 logarithm of these power spectra.

- Fig 5D (and others): when evaluating metamodel output accuracy, I think it would be useful to scale the error to the range of the simulations from the prior samples (i.e., training set), or was this already done?
This was not done. In our opinion, although it in many cases is easier to understand the errors in a relative scale, it is not necessarily so easy to do here. We can imagine a situation where e.g. 1 example in the training set has a particularly extreme value, which would then heavily skew the errors for the entire test data set if they were relative to the “output scale”.
Instead, we think the most intuitive way is just to present the absolute values for any errors, and also present the standard deviations of the metamodel outputs to get an idea of the scale.
- I think some figures can be labeled more informatively:
- Fig 5A&B can have subtitles, pop rate and LFP PSD, respectively, same for the two rows of 5D; the lines/shaded area in 5A&B are in general very difficult to see; 5C x-axis can be labeled test set index, and perhaps could be plotted as a cumulative distribution (so the transpose of the current graph) to make the same point but with a more standard plot;
Fixed. For the errors, we opted to just plot the distribution instead of the error for each particular test example.
- Fig 8A can have subtitles denoting spiking and LFP (and the two columns in Fig 8 in general), and red dot/line can be labeled as ground-truth in the figure; it’s a bit confusing that Fig8B breaks the 2-column pattern (but not sure if much can be done about it); lines in 8C are very difficult to see, and I think the black lines entirely block the shaded orange? Is it an issue that the two columns of 8B look almost identical? Is there some straightforward explanation for this? Typo in caption for 8D (“orange dashed line in C”)
We have added labels and titles, and made the black lines slightly thinner so it’s easier to see.
- I’m having a bit of a hard time understanding Fig 8D: is this the max distance of the metamodel mean from the posterior predictive simulations? Shouldn’t this be computed as a distance of the ground-truth simulation (i.e. the observation used for conditioning) from the posterior predictive simulations?
By plotting the distance from the metamodel output, we show that the posterior predictive distributions don’t significantly differ from what the metamodels “expect”. We think both approaches make sense, but agree that it might be better to plot the distance from the ground-truth, and have changed it.
- L460: full 10D pairplot would be nice to see in supplemental
We have added this
- L515-517: I think it would be clearer to use “conditioned on”, i.e., “…the coefficients for the samples from the posterior distribution conditioned on the population spiking activity,….and coefficients conditioned on the LFP”
We agree, and have changed it. 
Richard Gao, PhD
University of Tuebingen


Reviewer #3: The authors fit two statistical models ("metamodels"), to the Brunel network of excitatory and inhibitory LIF neurons. Some of the model parameters are treated as unknown and these unknown parameters are the input to the metamodel. The output of the metamodel are estimations of the power spectra of the population activities or of the LFP's. The metamodels are trained using supervised learning for many parameter sets from some domain of parameter space. The trained metamodels are tested on randomly sampled parameter combinations that lead to mostly asynchronous irregular spiking activity (parameters corresponding to (strong) synchronous activity are not considered both in the training and testing phase). The authors find an excellent performance of the metamodels both for the forward prediction of power spectra and for statistical inference of parameters given power spectrum estimates (inverse modeling). The parameter inference yields some interesting insights regarding the type of parameters and parameter combinations that can be well inferred, and the benefits of the inference on LFP data vs. population activity data (e.g. LFP data are advantageous for inference of synaptic connectivity). I found the paper important and interesting: the metamodel is a highly efficient model for the forward prediction of power spectra of LFPs or population activities given biophysically interpretable parameters, and inversely, the metamodel provides estimations of unknown biophysical parameters given power spectrum data. Both aspects are of high importance in neuroscience. The metamodelling framework is interesting because it combines the advantages of statistical models (efficiency, parameter inference) with the advantages of mechanistic network models (interpretability). The paper is scientifically sound and largely well written. The metamodel applies to state-of-the-art neural network models and LFP models in computational neuroscience and I expect the method to be highly useful at the interface of large-scale cortical recordings and network models of the corresponding circuits. The description of the method should be improved. Overall, I recommend publication in PLoS Computational Biology after the minor issues given below have been addressed.
We thank the reviewer for these positive remarks.
Minor issues
============

1) The introduction of the concept of metamodels is rather abstract/general and would benefit from a more concrete description (alongside the general concept). Figure 3 is very nice and already quite helpful in this respect, but only comes in Sec.3. For example, it should be made clear already in the introduction that, in the present paper, the input of the metamodel are some parameters of the network model and the output of the metamodel is some statistics (e.g. power spectra or distribution over power spectra) of some functions of network variables (e.g. LFP or population activities). It could be stressed that the metamodel does not generate realizations of population activities or LFPs, in fact there is no "time" in the metamodel, in contrast to the network model and other statistical models.
We have added additional clarification as to what exactly is being modelled in the introduction, as well as moved Figure 3 in order to give the reader an immediate overview.
We now write from lines 92: 
In the present work, we train metamodels to approximate the distribution over the power spectra of the population spiking activities and local field potential (LFP), based on the model parameters of the Brunel network [5], comprising an excitatory and an inhibitory population of recurrently connected point-neurons. The metamodels approximate the conditional probability distribution p(x|θ), where x is the power spectrum of either the population spiking activity or the LFP, and θ is the biological parameters of the spiking network. The local field potential, the low-frequency part of extracellular potentials, largely represents how synaptic inputs are processed in the dendrites [6, 16, 17], so we chose to include both signals as they in a sense represent the incoming and outgoing signals for a population, and it offers the opportunity to compare the information carried by the two different signals. Figure 1 shows an overview of the modelling scheme we employ.

2) Similar to point 1, I found the Methods part on Gaussian process regression rather abstract and incomprehensible to non-machine-learning experts. In Sec. 2.3, the quantities X, x and bold x seem to relate to the input, whereas the bold x has been defined as the output in the introduction (As I understand the bold x would be an estimate of the power spectra). Please, make sure that unique notations are used and all quantities are properly defined (x (not bold) does not seem to be defined, X should be defined more precisely, e.g. what is the dimension of the matrix, how is it constructed from n inputs (bold x?) of dimension d?). What is d in this paper? What is the "underlying function f(x)"? In line 200, if bold x denotes a vector (let's call it \vec{x}), (\vec{x}-\vec{x}')^2 is not clearly defined. Do you mean the norm squared (dot product)?
We agree. We have revised the whole section on GPR, and the notation is now consistent throughout the manuscript. 
3) What exactly is the output of the GPR? If it is the likelihood p(\vec{x}|\theta), how is it represented or computed (where is the equation for it)?
Yes, that is correct. We do not show an explicit expression for the predictions as the full equations would require a more detailed explanation of variational inference in the GP models and the numerical approximations that are used. We now give the overall equation for the model (eq. 15), but do not go into any more detail regarding the actual equations used for making predictions.
4) In line 269, the unnormalized posterior distribution is given in terms of the likelihood, which has been computed by the GPR. Why is it necessary to sample from this distribution? Isn't the unnormalized posterior distribution sufficient (e.g. to get the mean and standard deviation in the plots of this paper)?
We sample from the posterior distribution using MCMC. It’s not possible to get the mean or standard deviation of the posterior distribution (i.e. p(\theta | x_0)) directly from the metamodels, only the likelihood (i.e. p(x_0 | \theta)). 
5) Fig.5A,B: The 10 samples of the GPR (orange lines) are barely visible. On the other hand, I find it is not necessary and rather confusing to plot them. The output of the GPR is the distribution of the power spectrum, and hence it is sufficient to show the mean and the orange shaded area (two standard deviations). Therefore, I suggest to omit the orange lines, which will also lead to a better vivibility of the black lines. Furthermore, the authors write in line 385: "the smoothness of the power spectra is underestimated for lower frequencies, as can be observed by comparing the individual orange and black lines". I guess the mean power spectrum, which should be a much smoother function, is more relevant than individual samples because the mean power spectrum will provide an estimate of a perfectly averaged power spectrum in the network model (corresponding the averaging over infinitely many Hann windows in the Welch periodogram of an infinitely long simulation).
We agree, and have removed the individual lines, and plot only the mean and standard deviation instead.
6) For me the "power spectrum" is synonymous with power spectral density (PSD), which is defined by dividing by the window length T: PSD(f)=<[Psi]^*(f)[Psi](f)>/T (otherwise the power spectrum would scale with T). Why is the power spectrum defined without division by T? The scipy.signal.welch also provides the PSD that is rescaled by the window size. The PSD has also the advantage that the firing rate of neurons can be easily read-off from the high-frequency limit of the PSD of population activities (apart from a factor N). The PSD is not dimensionless, e.g. the PSD of population rates has units of 1/s, whereas without division by T it is dimensionless.
Thank you for pointing this out. This is an oversight on our part, and we have corrected the definition.
7) What is the unit of the LFP in this paper? The units of the corresponding power spectra seem to be missing in the plots unless the LFP has units of 1/time and hence its Fourier transform is unitless.
The units are mV. To help with the optimization of the metamodels, we take the logarithm of the PSD. That is also what we show in all the plots, and “hide” the units in the symbol for the PSD, since the logarithm assumes unitless inputs.
8) It is unclear how the method works outside the asynchronous irregular regime and where it fails. Could the method be used also for the synchronous regime?
In principle yes, but it would require scaling up the model, in particular the dimensionality of the latent space. We discuss it from line 523, where we say:
The simulations with strongly synchronous spiking activities were avoided since sharp peaks that shift continuously in frequency cannot be well approximated by a low-dimensional representation, and the low-rank approximation was necessary to keep the size of the DGP metamodel small. The low-rank approximation is a limiting factor for the metamodels, and ideally one would not have to limit the input domain in such a way. Increasing the dimensionality of the latent space is possible and would likely also have resulted in better accuracy, particularly with a larger training data set, but trade-offs have to be made with respect to the computational cost of training and evaluating the metamodels.
9) Are the rates nu in Fig.4 normalized by N? According to the definition of nu they are not, but in this case the single neuron rate would be nu/N which would result in extremely small rates. Furthermore, what exactly is nu here, is it a weighted sum of nu_E and nu_I? If nu is normalized then simply nu=nu_E=nu_I, but this is not the case if nu is not normalized.
Yes, in Figure 4 the rates are normalized by N, and only the excitatory population is shown. We have updated the figure text to reflect this.
10) There seems to be a numerical artifact in power spectrum at the highest frequency (strange decrease). This decrease should not be there because the PSD of the population activities saturates to a non-zero value (the saturation is already visible in the plots).
Thank you for pointing this out. We are not sure exactly what is causing it, but assume it has to do with Welch’s method as implemented in scipy. Since it only affects the two highest frequency channels and it doesn’t matter much for the metamodelling, we have not done anything about it, but it would also be possible to just exclude it.
11) In the asynchronous irregular regime, linear-response theory (especially the theory based on Lindner Phys Rev E 72, 061919, 2005) usually provides highly accurate analytical approximations of the PSDs (see e.g. Trousdale et al. PLoS Comput Biol. 2012, Deger et al. Phys. Rev E 2014, and the simpler but less accurate theories of Brunel J. Comput Neurosci. 2000 and Bos, Helias et al. PLoS Comput Biol. 2016). These analytical formulas should provide an efficient alternative to metamodels, which should be discussed in the paper.
We have added this to the discussion, from line 546. We now write:
Theoretical approaches based on linear-response theory can provide analytical approximations to the PSDs \cite{Lindner05,Trousdale12,Deger14,Bos16} in the asynchronous irregular regime. The theoretical models necessarily make assumptions about the underlying physics of the system, which also makes it possible to interpret them in ways that are not possible with purely data-driven methods. Data-driven methods are complementary to theoretical ones in the sense that they do not make any underlying assumption about the model, but comes at the price of interpretability and needing to run simulations to train the models.


Typos
=====
-caption Fig.5: set, . -> set.
Fixed
-caption Fig.5: errors are shown in standard deviations
See below
-Table 1: shaped-shaped -> alpha-shaped?
Fixed
-line 433: an an
Fixed
-line 451: membrane potential tau_m -> membrane time constant tau_m
Fixed
-caption Fig.8, last sentence: there should be two times a reference to panel C not B
Fixed
-caption Fig.5: errors are shown in standard deviations -> maybe a bit better: errors are shown in units of the standard deviations
No longer applicable to the new version of the figure.
